# Development and validation of the football emotion scale for Chinese youth players: a psychometric study

**DOI:** 10.3389/fpsyg.2026.1790940

**Published:** 2026-06-19

**Authors:** Ying Shuai, Shaoshen Wang, Garry Kuan, Bachok Norsa’adah, Yee Cheng Kueh

**Affiliations:** 1 School of Sports Management, Shandong Sport University, Shandong, China; 2 Biostatistics and Research Methodology Unit, School of Medical Sciences, Universiti Sains Malaysia, Kubang Kerian, Kelantan, Malaysia; 3 Exercise and Sports Science Programme, School of Health Sciences, Universiti Sains Malaysia, Kubang Kerian, Kelantan, Malaysia

**Keywords:** Chinese athletes, emotional assessment, football emotion scale, psychometric validation, youth football

## Abstract

**Background:**

Emotions play a crucial role in youth football performance and development, yet no validated instrument exists for measuring football-specific emotions among young players. This study aimed to develop and validate the Football Emotion Scale (FES) for Chinese youth football players.

**Methods:**

A cross-sectional survey was conducted among youth football players in China. The study included 492 participants in the exploratory factor analysis (EFA) phase (males 51.8%, females 48.2%) and 450 participants in the confirmatory factor analysis (CFA) phase (males 52.2%, females 47.8%). Participants were recruited through purposive sampling from 12 cities across Shandong Province. The scale’s psychometric properties were evaluated using Content Validity Index (CVI), Face Validity Index (FVI), EFA, CFA, Composite Reliability (CR), Average Variance Extracted (AVE), Cronbach’s alpha, and Intraclass Correlation Coefficient (ICC).

**Results:**

The EFA identified a six-factor structure explaining 67.027% of the total variance. The CFA confirmed this structure and supported a second-order model with two higher-order factors. Both models demonstrated excellent fit indices (RMSEA = 0.033, CFI = 0.971, TLI = 0.968, SRMR = 0.037). The scale showed good internal consistency (Cronbach’s alpha = 0.793–0.894) and test-retest reliability (ICC = 0.703–0.915). Construct validity was satisfactory, with composite reliability ranging from 0.793 to 0.894 and appropriate inter-factor correlations.

**Conclusion:**

The newly developed FES, consisting of 26 items, demonstrates robust psychometric properties and provides a valid and reliable tool for assessing football-specific emotions among Chinese youth players, facilitating research on emotional experiences and informing interventions to enhance player development and wellbeing.

## Introduction

Sports emotion has emerged as a critical area of study in athletic performance and player development, particularly in youth sports where emotional experiences can significantly impact both performance outcomes and overall wellbeing (Lazarus, 2000; Woodman et al., 2009). Research on emotion in sports has evolved substantially from its early focus primarily on anxiety to investigating a broader range of emotions and their complex dynamics in athletic performance (Furley et al., 2023; Jekauc et al., 2021). This evolution reflects a growing recognition that emotions play a multifaceted role in sports, encompassing physiological reactions, cognitive processes, behavioral tendencies, social functions, and performance outcomes.

Central to understanding these emotional dynamics are theoretical frameworks that link emotional states to performance outcomes. The Individual Affect-related Performance Zones (IAPZ) and the Individual Zone of Optimal Functioning (IZOF) models propose that athletes have optimal emotional “zones” where certain emotions enhance performance (Hanin, 2000; Kamata et al., 2002). Building on this foundation, contemporary understanding views emotions in sports as a complex integration of subjective experiences, psychophysiological expressions, biological reactions, and mental states (Schacter et al., 2011; Thatcher et al., 2011). These theoretical advances have paved the way for empirical investigations into how specific emotions influence athletic performance.

Research examining the relationship between emotions and sports performance has yielded significant insights. Santos (2017) reported that emotions such as anger, anxiety, and sadness were negatively associated with performance, while joy and vigor were linked to enhanced performance outcomes, although these findings should be interpreted with caution given the limited methodological detail provided. These patterns are consistent with broader evidence that positive emotions can enhance creativity, problem-solving skills, and decision-making in athletic contexts (McCarthy, 2011). At the team level, Campo et al. (2019) demonstrated that higher levels of positive emotions correlated with better team performance, highlighting the social function of emotions in sports settings.

In football specifically, emotions profoundly impact athlete performance, decision-making processes, and mental resilience (Correa, 2010). Studies have shown that positive emotions like excitement and confidence can enhance performance, while negative states such as anxiety may inhibit optimal functioning (Gheorghe et al., 2018). This emotional influence is particularly significant for young football players who are simultaneously managing the challenges of athletic development and adolescent psychological growth. Some preliminary evidence suggests that psychological interventions targeting rational thinking and emotional regulation may benefit young athletes’ mental preparation, although the methodological rigor of existing studies in this area remains limited (Gheorghe et al., 2019).

The field of emotion measurement in sports psychology has evolved considerably over the past decades. Several established emotion measurement tools have gained widespread recognition and application. The Positive and Negative Affect Schedule (PANAS) (Watson et al., 1988) represents a fundamental approach to emotion assessment, measuring general emotional states through two broad dimensions: positive and negative affect. The Profile of Mood States (POMS) (McNair et al., 1971) offers a more comprehensive assessment of mood variations, examining six distinct mood states including tension, depression, anger, vigor, fatigue, and confusion.

In sports-specific contexts, specialized measurement tools have been developed to address the unique emotional experiences of athletes. The Sport Emotion Questionnaire (SEQ) (Jones et al., 2005) was specifically designed to assess athletes’ emotional experiences across five dimensions: happiness, excitement, anxiety, dejection, and anger. The Brunel Mood Scale (BRUMS) (Terry et al., 1999), adapted from POMS, provides a more concise tool for assessing mood states in athletic contexts. These instruments have contributed significantly to our understanding of emotional experiences in sports settings.

However, these existing instruments present several limitations when applied to young football players, particularly in the Chinese context. The specific developmental stage of youth athletes is often overlooked in current measurement tools, which typically adopt an adult-oriented perspective. The particular emotional dynamics present in football, including the intense pressures of competition and the unique team dynamics, are not fully captured by general sports emotion scales. Moreover, cultural influences on emotion expression and experience are inadequately addressed in existing instruments, despite research highlighting the significance of cultural context in emotional expression and regulation (Duda and Allison, 1990).

The limitations of current measurement tools become particularly significant in the context of youth football, where players are increasingly subjected to adult-like professional pressures without adequate consideration for their developmental needs (Vaughan et al., 2022). Research in developmental sport psychology indicates that young athletes experience emotions differently from adults, characterized by greater emotional variability, heightened sensitivity to evaluative feedback from coaches and peers, and ongoing development of emotion regulation capacities (Crocker et al., 2015; Weinberg and Gould, 2023). These developmental characteristics necessitate age-appropriate approaches to emotional assessment rather than simply applying adult-oriented measurement tools.

Research further highlights that the pre-competitive and competitive phases in football present distinct emotional experiences requiring specific assessment approaches (Bueno and Souza, 2019). Athletes’ emotional states during these periods are significantly influenced by their roles and the competitive environment, necessitating measurement tools that can accurately capture these nuanced emotional experiences. The influence of cultural factors on emotional expression and regulation adds another layer of complexity, particularly in the Chinese sporting context.

Given these considerations, there is a clear need for a new measurement tool specifically designed to assess the emotional experiences of young Chinese football players. The theoretical framework underlying this study posits that emotions in youth football are organized along two dimensions: emotional valence (positive vs. negative) and situational context. Three contexts are distinguished: matches, involving competitive pressure and acute performance demands; training, characterized by repetitive skill development and daily coach-athlete interactions; and culture, encompassing broader identity-related processes such as players’ identification with football as a meaningful life domain (Hanin, 2000; Lazarus, 2000; Weinberg and Gould, 2023). This study aims to develop and validate a new Chinese language Football Emotion Scale (FES) that captures both positive and negative emotions across these three contexts, addressing the limitations of existing instruments while incorporating cultural and developmental considerations specific to young Chinese athletes.

Through rigorous statistical analysis and validation procedures, this study seeks to establish the psychometric properties of the FES, ensuring its reliability and validity for use in both research and practical applications. The development of this culturally sensitive and age-appropriate measurement tool will contribute to our understanding of emotional experiences in youth football while providing coaches and sports psychologists with a valuable instrument for assessing and monitoring young players’ emotional states.

## Materials and methods

### Study design

The current study employed a cross-sectional survey design conducted in two phases. The first phase for exploratory factor analysis (EFA) was carried out between May 2023 and July 2023, while the second phase for confirmatory factor analysis (CFA) was conducted between October 2023 and December 2023. For the EFA phase, 492 youth football players were recruited, while for the CFA phase, 450 youth football players participated. All participants were from National Youth Campus Football Featured Schools across 12 prefecture-level cities in Shandong Province, China. Additionally, a subset of 50 participants from the Shandong Luneng Taishan Football School in Weifang city was selected for the test-retest reliability assessment. These participants (mean age = 13.40 years, SD = 0.67; 50% male) completed the FES twice over a 14-day interval. The sample included players from all positions: defenders (36%), midfielders (32%), forward (20%), and goalkeepers (12%), distributed across Grade 1 (10%), Grade 2 (40%), and Grade 3 (50%) of junior high school.

The study followed a systematic data collection process through face-to-face questionnaire administration in quiet classroom settings. This approach was chosen over online surveys to ensure higher response rates and data quality, particularly considering the age group of the participants (Fowler, 2013). Research assistants were present during questionnaire completion to provide clarification if needed while maintaining standardized administration conditions across all participating schools.

A purposive multi-stage sampling approach was employed as it enabled the selection of information-rich cases while ensuring systematic participant selection based on specific criteria. This method was particularly appropriate for our study as it allowed access to participants within established institutional structures while maintaining the natural grouping of players within their training environments, thereby preserving the ecological validity of the research context (Cohen et al., 2002; Yin, 2015).

### Ethical approval

This study was conducted in accordance with the ethical principles outlined in the Declaration of Helsinki to ensure the highest standard of ethical research. Ethical approval was obtained from the Human Research Ethics Committee of Universiti Sains Malaysia (USM) under the approval code JEPeM Code: USM/JEPeM/22050288. Ethical guidelines were strictly followed throughout the research process to protect participants.

### Generation of items

The Football Emotion Scale (FES) items were generated through a systematic approach incorporating literature review and expert consultation. Initial item development began with a comprehensive review of existing literature on emotions in sports, particularly focusing on football-specific emotional experiences. This review included established measures such as the Sport Emotion Questionnaire (Jones et al., 2005), the Sport Competition Anxiety Test (Dunn and Dunn, 2001), the Positive and Negative Affect Schedule (Chen et al., 2016) and various other validated instruments in sports psychology.

The expert consultation process involved six experts: three senior youth football coaches with over 10 years of coaching experience at the provincial level, two sports psychology researchers specializing in adolescent athlete development, and one former professional football player with coaching qualifications. The consultation was conducted in three systematic stages. First, experts were provided with an overview of the study objectives and the concept of football emotions during an initial briefing. Following this, individual semi-structured interviews were conducted with each expert, focusing on key emotional experiences of adolescent football players, contextual factors influencing emotions in football, and suggestions for relevant emotional constructs to be included in the scale. All interviews were audio-recorded and transcribed verbatim for analysis, ensuring accurate capture of expert insights.

Based on the literature review and expert consultation, an initial pool of 42 items was developed, distributed across six theoretical dimensions: positive match emotions (7 items), negative match emotions (7 items), positive training emotions (7 items), negative training emotions (7 items), positive cultural emotions (7 items), and negative cultural emotions (7 items). These dimensions were established to comprehensively capture the emotional experiences specific to adolescent football players in the Chinese context. Each item was designed to reflect either positive or negative emotional experiences related to matches, training sessions, or football culture.

### Content validity, face validity, and pre-testing of the FES

Content validity was assessed through systematic evaluation by nine experts, including five youth football coaches with at least 5 years of experience and four sports psychology specialists. The experts rated each item’s relevance using a 4-point Likert scale (1 = Not Relevant to 4 = Highly Relevant). The Scale-level Content Validity Index (S-CVI/Ave) reached 0.87, approaching the desired threshold of 0.90. Among the initial 42 items, 36 achieved an Item-level Content Validity Index (I-CVI) ≥ 0.78, demonstrating good content validity for the majority of items (Polit et al., 2007).

Based on the CVI results and qualitative feedback from the expert panel, six items with I-CVI values below 0.78 were removed and replaced with six newly developed items, maintaining the 42-item structure. The S-CVI for Universal Agreement (S-CVI/UA) was 0.31, reflecting the stringent evaluation criteria applied by the nine-member expert panel. The removed items included those with ambiguous wording or limited relevance to the Chinese youth football context (e.g., Item 3: “Thinking a good performance is enough and injuries are unimportant,” I-CVI = 0.56; Item 11: “My normal performance will be affected by my opponent’s clinical performance,” I-CVI = 0.44). The replacement items were designed to better capture collaborative aspects of match emotions (e.g., “I enjoy the process of collaborating and competing with my teammates every time”), competition-related avoidance in negative match emotions (e.g., “Sometimes I want to avoid crucial matches”), training self-efficacy (e.g., “I believe that training will help me improve”), and training monotony (e.g., “Long-term monotonous training often makes me feel bored”). These modifications enhanced the scale’s comprehensiveness in capturing the emotional experiences specific to Chinese adolescent football players while maintaining strong content validity indices.

Pre-testing were conducted with 30 youth football players from Shandong Province in classroom settings. The results were predominantly positive, with participants reporting good comprehension of items and appropriate response options. The questionnaire completion time ranged from 15 to 25 min, which was deemed acceptable. No items were consistently flagged as unclear or difficult to understand, and there were no frequent incorrect responses, indicating overall clarity of the instrument.

The final version of the FES maintained its six-subscale structure: positive match emotion, negative match emotion, positive training emotion, negative training emotion, positive cultural emotion, and negative cultural emotion, with seven items in each subscale. All items were rated on a 5-point Likert scale ranging from 1 (Strongly Disagree) to 5 (Strongly Agree). Only minor formatting changes were implemented to enhance the questionnaire’s appearance and readability. The successful pre-testing phase, requiring minimal revisions, demonstrated the effectiveness of the expert review and content validity assessment processes in creating a comprehensible and culturally appropriate instrument for the target population.

### Statistical analysis

Data analysis was conducted using IBM SPSS Statistics version 28.0 for preliminary analyses and Mplus 8.0 for confirmatory factor analysis. The analysis process followed several systematic stages. Initial data screening included examination of descriptive statistics, with means, standard deviations, and frequency distributions calculated for all items. Item-total correlations and inter-item correlations were examined, with items showing correlations < 0.30 flagged for potential removal (DeVellis and Thorpe, 2021). The normality of item distributions was assessed through skewness and kurtosis values.

For the exploratory factor analysis (EFA), several assumption checks were performed. Univariate normality was assessed using histograms and Kolmogorov-Smirnov and Shapiro-Wilk tests. The positive definiteness of the covariance matrix was confirmed using principal component analysis (Abdi and Williams, 2010), and multicollinearity was assessed through examination of the inter-item correlation matrix, with correlations exceeding 0.85 flagged as potentially problematic (Brown, 2015). Additionally, tolerance and Variance Inflation Factor (VIF) values were examined to confirm the absence of multicollinearity. Sampling adequacy was verified through the Kaiser-Meyer-Olkin measure (threshold ≥ 0.7) and Bartlett’s test of sphericity (Hair et al., 2006). Principal Axis Factoring with Promax rotation was employed for factor extraction, as emotion factors were expected to correlate and Promax rotation is recommended for achieving a simpler and more interpretable factor structure when factors are correlated (Costello and Osborne, 2005). Factor retention was determined using multiple criteria: eigenvalues > 1, scree plot examination, and parallel analysis (Horn, 1965). Items with factor loadings below 0.40 were considered for removal (Tabachnick et al., 2013), and factor correlations above 0.85 were reviewed for potential multicollinearity issues (Brown, 2015).

For the confirmatory factor analysis (CFA), the robust maximum likelihood estimator (MLR) was employed for CFA estimation. This choice was based on several considerations: (a) comprehensive normality assessments, including Kolmogorov-Smirnov, Shapiro-Wilk, and Mardia’s multivariate kurtosis and skewness tests, confirmed significant deviations from normality in the data; (b) MLR provides robust standard errors and adjusted chi-square statistics that account for non-normality (Muthén and Muthén, 2017); and (c) simulation research has demonstrated that MLR performs comparably to WLSMV when items have five or more response categories and sample sizes are adequate (Li, 2016; Rhemtulla et al., 2012). Given that all FES items used a 5-point Likert scale and the CFA sample comprised 450 participants, MLR was considered an appropriate and defensible estimation method.

Model fit was evaluated using multiple indices: Comparative Fit Index (CFI ≥ 0.95 for good fit), Tucker-Lewis Index (TLI ≥ 0.95 for good fit), Root Mean Square Error of Approximation (RMSEA ≤ 0.06 for good fit), and Standardized Root Mean Square Residual (SRMR ≤ 0.08 for good fit) (Hu and Bentler, 1999; Kline, 2023). Factor loadings were assessed with values ≥ 0.50 considered satisfactory (Hair et al., 2010). Construct reliability (CR) was calculated with values > 0.70 considered reliable, and internal convergence was assessed through average variance extracted (AVE), with values > 0.50 considered satisfactory (Fornell and Larcker, 1981). Discriminant validity was evaluated using the Fornell-Larcker criterion, which requires the square root of AVE for each construct to exceed its correlations with other constructs. Model modifications were made based on modification indices only when theoretically justified (Byrne, 2010). Internal consistency reliability was assessed using Cronbach’s alpha, with values > 0.70 considered acceptable (Nunnally and Bernstein, 1994). For test-retest reliability, Intraclass Correlation Coefficient (ICC) was calculated using two-way mixed-effects models with absolute agreement. ICC values were interpreted as: < 0.50 poor, 0.50–0.75 moderate, 0.75–0.90 good, and > 0.90 excellent (Koo and Li, 2016).

## Results

### Descriptive statistics

A total of 942 youth football players participated in this study, with 492 participants in the exploratory factor analysis (EFA) phase and 450 in the confirmatory factor analysis (CFA) phase. The mean age was 13.11 years (SD = 0.04) for the EFA sample and 12.94 years (SD = 0.78) for the CFA sample. In the EFA sample, gender distribution was relatively balanced, with 255 males (51.8%) and 237 females (48.2%). Participants were distributed across three grades of junior high school, with the majority in Grade 2 (44.1%), followed by Grade 1 (30.5%) and Grade 3 (25.4%). Regarding playing positions, 198 participants (40.2%) were defenders, while the remaining positions were relatively evenly distributed among forward (25.0%), midfielders (25.2%), and goalkeepers (9.6%). The CFA sample showed a similar demographic composition, though with some variations in distribution. The CFA sample showed a similar demographic composition, with 235 males (52.2%) and 215 females (47.8%). Playing positions were more evenly distributed in the CFA sample, with 177 defenders (39.3%), 118 midfielders (26.2%), 113 forward (25.1%), and 42 goalkeepers (9.3%). Participants were recruited from 12 cities across Shandong Province (Table 1).

**TABLE 1:** Demographic characteristics of EFA and CFA participants.

Category	Name	EFA (492)		CFA (450)	
		*n*(%)	Mean (SD)	*n*(%)	Mean (SD)
Age			13.11 (0.04)		12.94 (0.78)
City	Binzhou	32 (6.5)		19 (4.2)	
	Dongying	31 (6.3)		33 (7.3)	
	Heze	18 (3.7)		18 (4.0)	
	Jinan	44 (8.9)		37 (8.2)	
	Jining	35 (7.1)		28 (6.2)	
	Liaocheng	43 (8.7)		35 (7.8)	
	Linyi	86 (17.5)		59 (13.1)	
	Qingdao	44 (8.9)		41 (9.1)	
	Tai’an	41 (8.3)		38 (8.4)	
	Weifang	20 (4.1)		69 (15.3)	
	Zaozhuang	28 (5.7)		28 (6.2)	
	Zibo	70 (14.2)		45 (10.0)	
Gender	Male	255 (51.8)		235 (52.2)	
	Female	237 (48.2)		215 (47.8)	
Grade	Grade 1 of junior high school	150 (30.5)		155 (34.4)	
	Grade 2 of junior high school	217 (44.1)		237 (52.7)	
	Grade 3 of junior high school	125 (25.4)		58 (12.9)	
Position	Forward	123 (25.0)		113 (25.1)	
	Midfielder	124 (25.2)		118 (26.2)	
	Defender	198 (40.2)		177 (39.3)	
	Goalkeeper	47 (9.6)		42 (9.3)	

EFA, exploratory factor analysis; CFA, confirmatory factor analysis; SD, standard deviation; *n*, number.

### EFA results of the FES

Prior to factor extraction, multicollinearity assessment confirmed that no inter-item correlations exceeded 0.85, and all items demonstrated acceptable tolerance (> 0.1) and VIF (< 10) values, indicating that multicollinearity did not unduly influence the factor structure. The Kaiser-Meyer-Olkin test (0.906) and Bartlett’s test of sphericity (*p* < 0.001) indicated the suitability of the data for factor analysis. Principal axis factoring with Promax rotation identified six factors explaining 67.027% of the total variance. Through iterative analysis, 16 items were eliminated based on factor loadings below 0.40 and cross-loadings, resulting in a 26-item structure. The complete content of all 26 retained items is presented in Supplementary Appendix 1.

The six extracted factors were: football positive match emotions (6 items, loadings 0.682–0.894), football negative match emotions (4 items, loadings 0.647–0.792), football positive training emotions (3 items, loadings 0.613–0.727), football negative training emotions (4 items, loadings 0.680–0.829), football positive culture emotions (4 items, loadings 0.556–0.765), and football negative culture emotions (5 items, loadings 0.689–0.781). Internal consistency reliability was strong across all factors, with Cronbach’s alpha coefficients ranging from 0.788 to 0.894. The first two factors explained the largest proportion of variance (46.30%), with the remaining factors contributing incrementally to the total explained variance (Table 2a). The factor correlation matrix from the EFA is presented in Table 2b, with all inter-factor correlations below the 0.85 threshold.

**TABLE 2a:** Items descriptive statistics, exploratory factor analysis.

Factor	Item content	Mean (SD)	Factor loading					
			1	2	3	4	5	6
Football positive match emotions	FES1	3.69 (0.91)	**0.781**					
	FES2	3.71 (0.92)	**0.729**					
	FES3	3.08 (1.32)	−					
	FES4	3.72 (0.90)	**0.717**					
	FES5	3.65 (0.95)	**0.721**					
	FES6	3.76 (0.94)	**0.682**					
	FES7	3.89 (0.99)	**0.894**					
Football negative match emotions	FES8	1.96 (1.04)				−		
	FES9	2.31 (1.17)				−		
	FES10	3.48 (1.18)				−		
	FES11	2.20 (0.91)				**0.792**		
	FES12	1.54 (1.04)				**0.722**		
	FES13	1.82 (0.88)				**0.658**		
	FES14	2.37 (0.82)				**0.647**		
Football positive training emotions	FES15	4.04 (0.97)						**0.727**
	FES16	3.81 (1.00)						**0.613**
	FES17	3.92 (0.97)						**0.709**
	FES18	3.21 (1.13)			−			
	FES19	3.65 (1.08)			−			
	FES20	3.32 (1.05)			−			
	FES21	3.57 (1.07)			−			
Football negative training emotions	FES22	2.51 (1.14)			−			
	FES23	2.01 (1.01)			−			
	FES24	2.09 (0.98)			**0.814**			
	FES25	2.01 (1.04)			**0.680**			
	FES26	1.70 (0.88)			−			
	FES27	2.04 (0.95)			**0.829**			
	FES28	2.16 (0.95)			**0.771**			
Football positive culture emotions	FES29	4.11 (0.98)					**0.556**	
	FES30	3.92 (1.03)					**0.720**	
	FES31	4.02 (0.94)					**0.724**	
	FES32	3.98 (0.97)					**0.765**	
	FES33	3.95 (0.98)		−				
	FES34	3.68 (1.28)		−				
	FES35	4.18 (0.97)		−				
Football negative culture emotions	FES36	1.9 (0.96)		**0.711**				
	FES37	1.89 (1.00)		**0.689**				
	FES38	1.69 (0.93)		**0.772**				
	FES39	1.73 (0.99)		**0.781**				
	FES40	1.74 (0.94)		**0.716**				
	FES41	2.27 (1.20)		−				
	FES42	1.36 (0.77)		−				
	Eigenvalue		6.973	5.065	1.566	1.524	1.264	1.035
	Variance explained (%)		26.819	19.481	6.023	5.860	4.863	3.982
	Cumulative variance (%)		26.819	46.300	52.323	58.183	63.045	67.027
	Cronbach alpha		0.894	0.806	0.788	0.872	0.804	0.863

SD, standard deviation. Loadings shown are from the pattern matrix obtained through Principal Axis Factoring with Promax rotation. Only defining loadings (≥ 0.40) are presented for clarity. Bold values indicate the defining factor loadings retained for each factor. All cross-loadings for retained items ranged from −0.136 to 0.119, well below the 0.40 threshold. Items marked with “−” were removed due to factor loadings below 0.40 or presence of cross-loadings.

**TABLE 2b:** Factor correlation matrix from EFA.

Factor	FPME	FNCE	FNTE	FNME	FPCE	FPTE
*FPME*	1	1	1	1	1	1
*FNCE*	−0.124					
*FNTE*	−0.157	0.614				
*FNME*	−0.077	0.547	0.581			
*FPCE*	0.579	−0.128	−0.117	−0.118		
*FPTE*	0.576	−0.215	−0.115	−0.086	0.602	

Extraction Method: Principal Axis Factoring; Rotation Method: Promax with Kaiser Normalization. FPME, Football Positive Match Emotion; FNCE, Football Negative Culture Emotion; FNTE, Football Negative Training Emotion; FNME, Football Negative Match Emotion; FPCE, Football Positive Culture Emotion; FPTE, Football Positive Training Emotion. All inter-factor correlations were below the 0.85 threshold, supporting the distinctiveness of the six factors.

### CFA results of the FES

**FIGURE 1:**
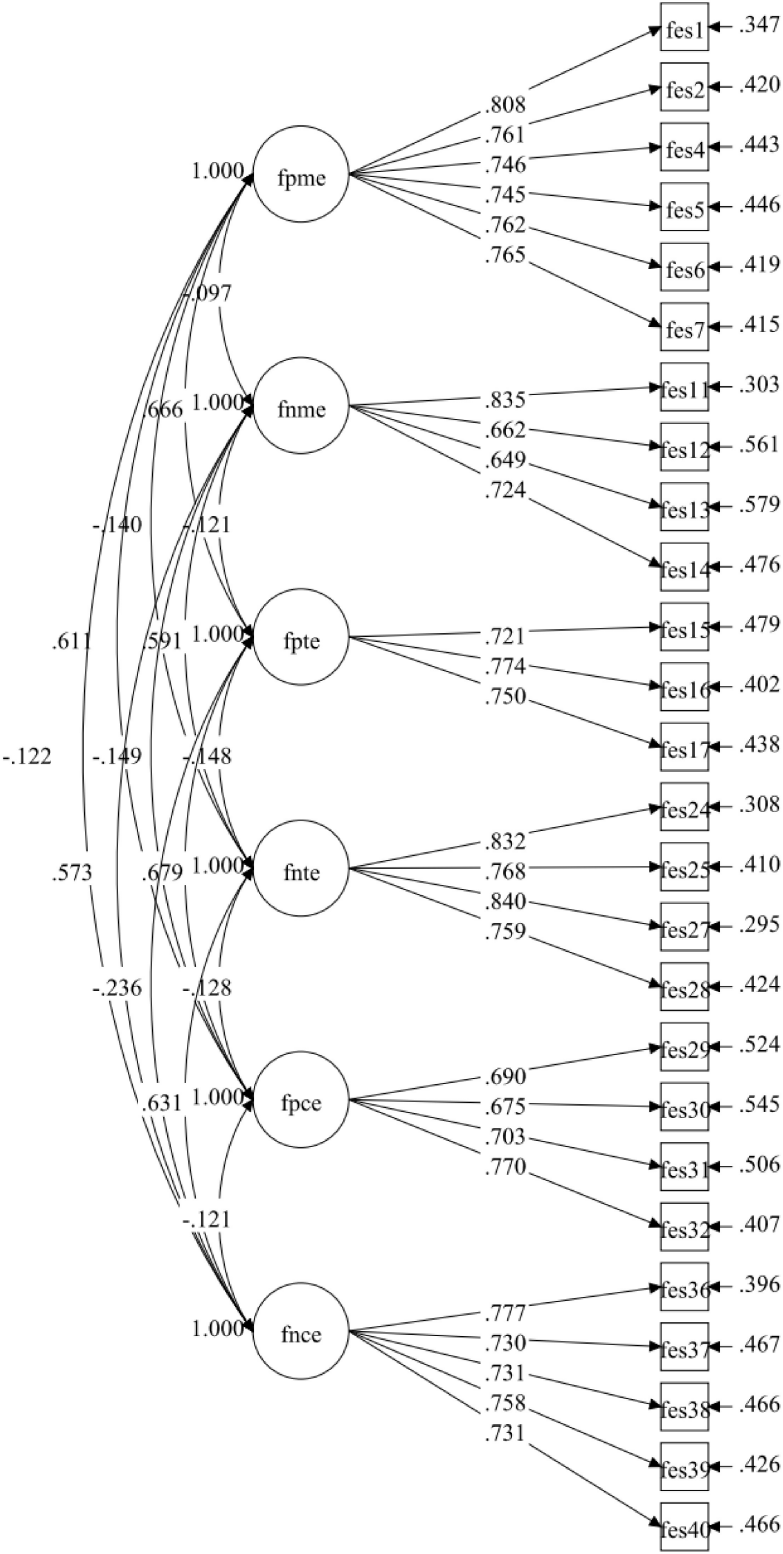
CFA diagram of the FES-C initial model. FPME, Football Positive Match Emotion; FNME, Football Negative Match Emotion; FPTE, Football Positive Training Emotion; FNTE, Football Negative Training Emotion; FPCE, Football Positive Culture Emotion; FNCE, Football Negative Culture Emotion. Latent factor variances are fixed at 1.0 for identification purposes in the standardized solution. The complete inter-factor correlation matrix is presented in Table 4.

**FIGURE 2:**
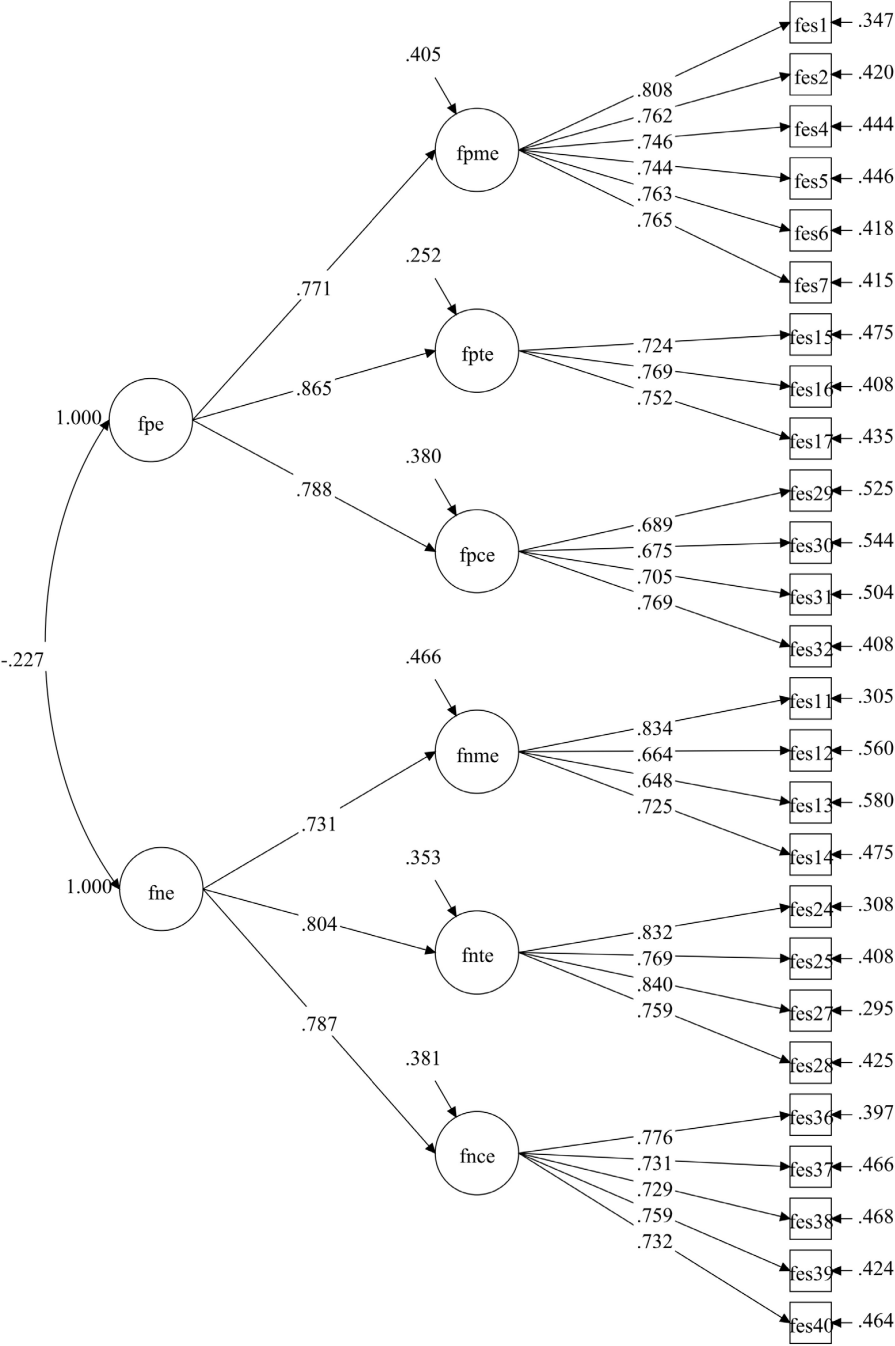
CFA diagram of the FES-C second order model. FPE, Football Positive Emotion; FNE, Football Negative Emotion; FPME, Football Positive Match Emotion; FNME, Football Negative Match Emotion; FPTE, Football Positive Training Emotion; FNTE, Football Negative Training Emotion; FPCE, Football Positive Culture Emotion; FNCE, Football Negative Culture Emotion.

The six-factor structure identified in the EFA was tested through confirmatory factor analysis with a new sample (*n* = 450). Two models were evaluated: a first-order model with six correlated factors and a second-order model with two higher-order factors (Football Positive Emotion and Football Negative Emotion). The model fit indices are presented in Table 3. The factor loadings in the first-order model ranged from 0.649 to 0.840 across all dimensions. The Football Positive Match Emotion dimension showed strong loadings (0.745–0.808), as did Football Negative Training Emotion (0.759–0.840) and Football Negative Culture Emotion (0.730–0.777) (Figure 1). In the second-order model, loadings of the first-order factors onto their respective higher-order factors were substantial (0.771–0.865 for positive emotions; 0.731–0.804 for negative emotions), supporting the hierarchical structure (Figure 2).

**TABLE 3:** Summary for FES-C model fit indices.

CFA model	RMSEA (90% CI)	CFI	TLI	SRMR
Model-1	0.033 (0.026, 0.040)	0.971	0.967	0.035
Model-2	0.033 (0.026, 0.039)	0.971	0.968	0.037

Model-2 represents the second-order CFA model where higher-order factors are modeled to explain the relationships between the first-order factors. Specifically, this model includes two second-order factors: FPE (Football Positive Emotion) and FNE (Football Negative Emotion), which are hypothesized to account for the correlations among their respective first-order factors (FPME, FPTE, FPCE for FPE and FNME, FNTE, FNCE for FNE).

### Construct validity of the FES

The construct validity of both the first-order and second-order models was examined through composite reliability (CR), average variance extracted (AVE), and factor correlations. CR and AVE values were used to assess internal convergence among items within each factor, while the Fornell-Larcker criterion was applied to evaluate discriminant validity between factors. In the first-order model, all factors demonstrated good composite reliability, ranging from 0.793 to 0.894. The AVE values ranged from 0.505 to 0.641, exceeding the recommended threshold of 0.50, indicating satisfactory internal convergence within each factor. Factor correlations ranged from −0.236 to 0.679, all below the 0.85 threshold, and the square root of AVE for each factor exceeded its correlations with other factors, supporting discriminant validity according to the Fornell-Larcker criterion (Table 4). In the second-order model, both higher-order factors demonstrated good internal convergence, with CR values of 0.850 and 0.818 for Football Positive Emotion (FPE) and Football Negative Emotion (FNE), respectively. The AVE values were 0.655 for FPE and 0.600 for FNE, both exceeding the 0.50 threshold. The correlation between FPE and FNE was −0.227 (*p* < 0.05), and the square roots of AVE (0.809 for FPE and 0.775 for FNE) were greater than their factor correlation, further supporting discriminant validity between the two higher-order factors (Table 5).

**TABLE 4:** Correlation matrix and construct validity of the FES-C (first-order model).

Construct	CR.	AVE.	FPME	FNME	FPTE	FNTE	FPCE	FNCE
FPME	0.894	0.585	0.765	0.721	0.749	0.801	0.710	0.746
FNME	0.811	0.520	−0.097					
FPTE	0.793	0.560	0.666*	−0.121*				
FNTE	0.877	0.641	−0.140*	0.591*	−0.148*			
FPCE	0.803	0.505	0.611*	−0.149*	0.679*	−0.128*		
FNCE	0.862	0.556	−0.122*	0.573*	−0.236*	0.631*	−0.121*	

*p < 0.05; CR, Composite Reliability; AVE, Average Variance Extracted; FPME, Football Positive Match Emotion; FNME, Football Negative Match Emotion; FPTE, Football Positive Training Emotion; FNTE, Football Negative Training Emotion; FPCE, Football Positive Culture Emotion; FNCE, Football Negative Culture Emotion. Diagonal elements (in bold) represent the square root of AVE, while off-diagonal elements represent the correlations between constructs.

**TABLE 5:** Correlation matrix and construct validity of the FES-C (second-order model).

Construct	CR.	AVE.	FPE	FNE
FPE	0.850	0.655	0.809	
FNE	0.818	0.600	−0.227*	0.775

*p < 0.05; CR, Composite Reliability; AVE, Average Variance Extracted; FPE, Football Positive Emotion; FNE, Football Negative Emotion. Diagonal elements (in bold) represent the square root of AVE, while off-diagonal elements represent the correlations between constructs.

### Test-retest reliability of the FES

Test-retest reliability was evaluated on a subset of 50 participants from the Shandong Luneng Taishan Football School who completed the FES twice over a 14-day interval. For the first-order model, the intraclass correlation coefficients (ICC) demonstrated strong temporal stability across all six dimensions, ranging from 0.703 to 0.876. Football Positive Match Emotion (ICC = 0.871), Football Positive Culture Emotion (ICC = 0.853), and Football Negative Culture Emotion (ICC = 0.876) showed particularly high stability (Table 6). The second-order model also exhibited excellent test-retest reliability, with ICC values of 0.915 for Football Positive Emotion and 0.805 for Football Negative Emotion. These results indicate that the FES demonstrates robust temporal stability at both the first-order and second-order factor levels (Table 7).

**TABLE 6:** Intraclass correlation coefficient (ICC) for the FES-C model 1.

Dimension	ICC
Football positive match emotion	0.871
Football negative match emotion	0.703
Football positive training emotion	0.822
Football negative training emotion	0.820
Football positive culture emotion	0.853
Football negative culture emotion	0.876

ICC, intraclass correlation coefficient.

**TABLE 7:** Intraclass correlation coefficient (ICC) for the FES-C second order model.

Dimension	ICC
Football positive emotion	0.915
Football negative emotion	0.805

ICC, intraclass correlation coefficient.

**FIGURE 3:**
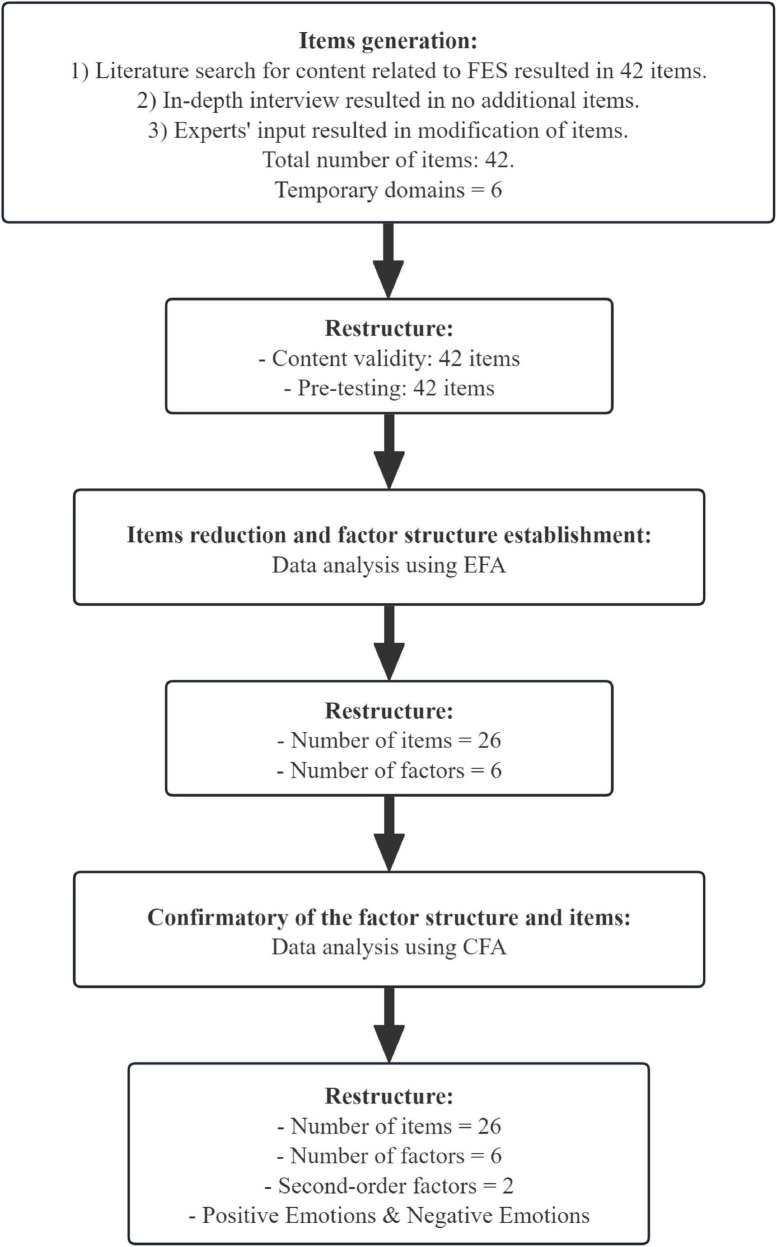
Summary of FES development process.

The FES questionnaire development process is presented in Figure 3.

## Discussion

This study developed and validated the Football Emotion Scale (FES) for Chinese youth football players, demonstrating robust psychometric properties and a theoretically sound factor structure. The results provide several key insights into the measurement of sports-specific emotions and their implications for youth football development.

The six-factor structure of the FES captured both positive and negative emotional experiences across three distinct football contexts: matches, training sessions, and football culture. This structure is grounded in the theoretical rationale that emotions in youth football are shaped not only by their valence (positive or negative) but also by the situational context in which they arise. Match emotions reflect the competitive pressures and social evaluation inherent in game situations; training emotions capture the motivational and affective responses to daily practice routines; and cultural emotions represent broader identification with and attitudes toward football as a meaningful life domain (Hanin, 2000; Lazarus, 2000). The excellent model fit indices for both the first-order and second-order models (RMSEA = 0.033, CFI = 0.971, TLI = 0.968, SRMR = 0.037) provide empirical support for this theoretical framework, and the development of the FES addresses a gap in sports psychology measurement noted by Crocker et al. (2015).

The emergence of two higher-order factors—Football Positive Emotion and Football Negative Emotion—in the second-order model aligns with established theoretical frameworks distinguishing positive and negative affect (Fredrickson, 2001; Watson et al., 1988). This hierarchical structure suggests that while youth football players experience distinct emotions across different contexts, these specific emotional experiences are organized under broader positive and negative affective dimensions. The successful validation of both models provides flexibility in application: the first-order model is suited for research examining context-specific emotional dynamics (e.g., how training emotions differ from match emotions), while the second-order model offers a more parsimonious framework for studies investigating broader emotional patterns or for use in applied settings where brevity is important (Fletcher and Sarkar, 2012).

The scale demonstrated strong psychometric properties across multiple indicators. Internal consistency was good to excellent (Cronbach’s alpha = 0.793–0.894), comparable to established instruments such as the SEQ (Jones et al., 2005) and PANAS (Watson et al., 1988). Test-retest reliability over a 14-day interval was adequate to excellent (ICC = 0.703–0.915), supporting the temporal stability of the measured constructs. These reliability indices suggest that the FES is suitable for use in both cross-sectional research and longitudinal studies tracking emotional changes in youth football development (Koo and Li, 2016).

Construct validity was supported through multiple lines of evidence. The CR values (0.793–0.894) and AVE values (0.505–0.641) indicated satisfactory internal convergence among items within each factor. Discriminant validity was confirmed through the Fornell-Larcker criterion, with the square root of AVE for each factor exceeding its correlations with other factors, and all inter-factor correlations remaining below the 0.85 threshold. These findings provide evidence for the structural validity of the FES, confirming that the six factors represent distinct yet theoretically related emotional constructs.

Several limitations should be considered when interpreting these results. First, the sample was limited to youth football players in Shandong Province, which may affect generalizability to other regions, age groups, or competitive levels. Although Shandong Province encompasses diverse urban and rural settings across 12 cities, replication in other provinces and cultural contexts is warranted. Shandong Province is one of China’s most developed regions for campus football, and the emotional experiences of youth players in this context may differ from those in provinces with less established football infrastructure or different socio-cultural environments. Additionally, the purposive multi-stage sampling approach, while appropriate for accessing participants within established institutional structures, limits the ability to draw population-level inferences. Future studies should employ probability-based sampling across multiple provinces to enhance the representativeness and generalizability of the findings. Second, while the cross-sectional design established the scale’s initial psychometric properties, longitudinal studies are needed to examine the stability of football-related emotions over time and the scale’s sensitivity to change following interventions. Third, while the current study tested a first-order six-factor model and a second-order two-factor model, alternative model specifications such as bifactor models were not examined. The second-order model was selected based on its alignment with established emotion theory distinguishing positive and negative affect (Watson et al., 1988), and the six-factor first-order model was retained as it directly reflected the empirical EFA results. Future research with larger item pools could explore bifactor specifications to further disentangle the contributions of emotional valence and situational context to item variance (Reise et al., 2016). Fourth, while construct validity was supported through factorial structure, internal convergence (AVE > 0.50), and discriminant validity (Fornell-Larcker criterion), the current study did not include a comprehensive examination of convergent validity with external criterion measures. Future studies should examine associations between the FES and other established sport emotion measures (e.g., the SEQ), behavioral indicators such as observed performance, or coach ratings to further strengthen criterion validity evidence. Additionally, future validation using WLSMV estimation could serve as a robustness check for the CFA results obtained with MLR.

Future research should examine the scale’s psychometric properties across different cultural contexts and age groups to enhance its generalizability. Longitudinal studies investigating the scale’s predictive validity for performance outcomes and psychological wellbeing would further establish its utility in youth sports development. These investigations would strengthen the evidence base for using the FES in both research and applied settings, ultimately contributing to better understanding and support of young athletes’ emotional experiences in football.

## Conclusion

This study developed and validated the Football Emotion Scale (FES), establishing its psychometric properties through a systematic process of item development, content validation, and construct validation. The final instrument comprises six first-order factors (match, training, and culture-related emotions, both positive and negative) and two second-order factors (positive and negative emotions), providing a comprehensive assessment of football-specific emotional experiences among youth players. The development and validation of the FES advances our understanding of emotional assessment in youth sports by providing a psychometrically robust tool that captures the unique emotional experiences in football. The scale’s strong validity evidence and reliability coefficients suggest it can effectively measure both specific emotional dimensions and broader emotional patterns, contributing to the theoretical framework of emotions in sports psychology. These findings have important implications for both research and practical applications in youth football development programs.

## Data Availability

The raw data supporting the conclusions of this article will be made available by the authors, without undue reservation.
