# Supplementary material for: Development and validation of the football emotion scale for Chinese youth players: a psychometric study
Source: Front Psychol. 2026 Jun 19;17:1790940. doi: 10.3389/fpsyg.2026.1790940 (PMC13328403; doi:10.3389/fpsyg.2026.1790940)
Supplement: Supplementary file 1 [file Table_1.DOCX]

**Appendix A: English version: Football Emotion Scale**

Please carefully read each sentence below and evaluate the degree to which the description matches your own situation. Please use 1-5 to indicate the degree of matching you believe, with a higher value indicating a better fit.

**1 is completely inconsistent, 2 is not very consistent, 3 cannot be determined, 4 is relatively consistent, and 5 is completely consistent**

| Your emotional description | 1 | 2 | 3 | 4 | 5 | Status |
| --- | --- | --- | --- | --- | --- | --- |
| 1. **I feel happy when competing with my competitors** |  |  |  |  |  |  |
| 1. **The more crucial the competition is, the more excited I get** |  |  |  |  |  |  |
| 1. I enjoy the process of collaborating and competing with my teammates every time |  |  |  |  |  | Removed |
| 1. I am confident of achieving success in the competition |  |  |  |  |  |  |
| 1. **I look forward to strong opponents appearing in the competition** |  |  |  |  |  |  |
| 1. **I am so drowned in the game, that sometimes I forget the passage of time** |  |  |  |  |  |  |
| 1. **I am eager for every competition to come** |  |  |  |  |  |  |
| 1. Sometimes I want to avoid (escape) some crucial (important) matches |  |  |  |  |  | Removed |
| 1. Tense about the upcoming competition |  |  |  |  |  | Removed |
| 1. The change in weather will affect my performance during the competition |  |  |  |  |  | Removed |
| 1. **When I compete, I worry about making mistakes** |  |  |  |  |  |  |
| 1. **When waiting to take the stage, my breathing becomes rapid** |  |  |  |  |  |  |
| 1. **I would beat myself up for losing the occasional competition** |  |  |  |  |  |  |
| 1. **I feel distracted and can't sit still when watching our team compete** |  |  |  |  |  |  |
| 1. **Time always flies when I'm training.** |  |  |  |  |  |  |
| 1. **When I train, I feel strong and energised.** |  |  |  |  |  |  |
| 1. **Training is interesting and enjoyable for me** |  |  |  |  |  |  |
| 1. I believe that training will help me improve |  |  |  |  |  | Removed |
| 1. I do not feel good if I miss a day of training |  |  |  |  |  | Removed |
| 1. I exercise outside of team practices |  |  |  |  |  | Removed |
| 1. When I train, I forget everything around me |  |  |  |  |  | Removed |
| 1. I feel exhausted when I think about having to face another day on the training |  |  |  |  |  | Removed |
| 1. I feel emotionally drained from my training |  |  |  |  |  | Removed |
| 1. **Long term monotonous training often makes me feel bored** |  |  |  |  |  |  |
| 1. **I feel like I have no choice about training; Coaches make me do it** |  |  |  |  |  |  |
| 1. I don't have a clear training goal myself, as long as I complete the task |  |  |  |  |  | Removed |
| 1. **I feel bored in my daily training** |  |  |  |  |  |  |
| 1. **During the training process, I occasionally check my watch** |  |  |  |  |  |  |
| 1. **Football is largely a source of strength and comfort** |  |  |  |  |  |  |
| 1. **Football is my main motivation** |  |  |  |  |  |  |
| 1. **Getting to know my favourite football icons has given me a lot of pleasure** |  |  |  |  |  |  |
| 1. **Football competition is more exciting than other sports competition** |  |  |  |  |  |  |
| 1. I feel sad when the team has to sack some people because of changes |  |  |  |  |  | Removed |
| 1. I'm a big fan of a certain football team |  |  |  |  |  | Removed |
| 1. I am proud to be a footballer |  |  |  |  |  | Removed |
| 1. **Playing football has had a negative impact on my physical and mental health** |  |  |  |  |  |  |
| 1. **Because of playing football, I encountered difficulties in my studies** |  |  |  |  |  |  |
| 1. **Pessimistic and disappointed with my football future** |  |  |  |  |  |  |
| 1. **I am not interested in anything else about football except for my own game training** |  |  |  |  |  |  |
| 1. **Maybe at some point I'll give up football** |  |  |  |  |  |  |
| 1. I will imitate the bad behavior or hobbies of my football idol |  |  |  |  |  | Removed |
| 1. I chose football passively because I didn't have any other options |  |  |  |  |  | Removed |

Note: Items in bold were retained in the final 26-item scale (6 subscales: Positive Match Emotion = FES1, 2, 4, 5, 6, 7; Negative Match Emotion = FES11, 12, 13, 14; Positive Training Emotion = FES15, 16, 17; Negative Training Emotion = FES24, 25, 27, 28; Positive Culture Emotion = FES29, 30, 31, 32; Negative Culture Emotion = FES36, 37, 38, 39, 40). Items in regular font were removed during exploratory factor analysis due to factor loadings below 0.40 or cross-loadings.
